# Highlights from the Romanian National Congress of Oncology: challenges in oncology in 2020, 15-17 October 2020 Poiana Brasov, Romania

**DOI:** 10.3332/ecancer.2021.ed109

**Published:** 2021-03-01

**Authors:** Dana Stanculeanu, Claudia Burz

**Affiliations:** 1 President, Romanian National Society of Medical Oncology; Department of Oncology, Faculty of General Medicine, ‘Carol Davila’ University of Medicine and Pharmacy, Bucharest; Department of Medical Oncology, ‘Prof. Dr Alexandru Trestioreanu’ Institute of Oncology, Bucharest 022338, Romania; 2 President, Society of Radiotherapy and Medical Oncology; Department of Oncology, Faculty of General Medicine, ‘Iuliu Hațieganu’ University of Medicine and Pharmacy, Cluj-Napoca; Department of Medical Oncology, ‘Prof. Dr Ion Chiricuta’ Institute of Oncology, Cluj-Napoca 400015, Romania

**Keywords:** congress, oncology societies, challenges, virtual

## Abstract

In a fast-moving world of change and adaptation, the first conference of the three oncology societies from Romania had as its main topic “The challenges in oncology in 2020”. It proved to successfully replace a traditional conference by a virtually held event. Also, for the first time it united all oncology and radiotherapy societies under the same umbrella connected by the same aspirations of collaboration and sharing innovative ideas and progress for better cancer care and outcome in Romania.

The Romanian National Congress of Oncology was held in October 2020 in Poiana Brasov and its theme was current oncology challenges. The topic was chosen after careful consideration of the present coronavirus pandemic and the difficulties of accessing the healthcare system for oncology patients. The participation was virtual, using an online platform. For the first time, all three major national oncology societies (the Medical Oncology Society, Radiotherapy Society and Radiotherapy and Oncology Society) decided to join together and hold a common national congress. The conference enjoyed the largest international participation ever recorded for a Romanian oncological conference, bringing together more than 600 delegates with the vast majority from medical oncology, followed by radiotherapy, genetics, imaging and anatomical pathology with vibrant discussions over 3 full days.

The official opening enjoyed an eminent international participation: ASCO president Prof. Dr. Clifford Hudis, the president of the American Association of Physicists Prof. Dr. Mohammed Huq Saiful and the president of the European Society for Radiotherapy and Oncology, Prof. Dr. Ben Slotman. The President of the Romanian Academy, Prof. Ioan Aurel Pop and a number of distinguished vice-chancellors of Romanian Universities and representatives of the Romanian Ministry of Health also addressed the audience.

Each day more than 500 specialists connected online with dynamic interactions challenging each other regarding the best options for patients and recent advances in oncology while sharing and providing solutions for access for oncology patients

Key themes covered during the conference included: fundamental research, oncology education and training, radiotherapy post ASTRO, support programs, challenges and opportunities, research conducted by residents, and a number of dedicated sessions for each group of pathology.

The best basic research presentations received prizes and a lot of interest was shared to the discussion of the European Cancer Plan and the opportunities for countries in developing a comprehensive package for patients with cancer.

The Congress included special sessions on innovation in oncology, a session on original papers and research, as well as planning and debating sessions on oncology needs and future priorities for Romanian patients with cancer. There were many sessions focusing on the latest therapeutic advances and predictions for the future of cancer treatment. Special attention was given to eposters with many original and innovative presentations.

Current standards of care for Romanian patients in public and private institutions were both challenged and celebrated. New and innovative immunotherapies also came into the spotlight. The need for multidisciplinary tumour boards was highlighted and it was proposed that each regional cancer centre should institute a regional board. A number of sessions were dedicated to nurses, physicists, social workers, psychologists, NGOS and pharmaceutical companies’ presentations.

The meeting, held over three days, attracted numerous high calibre speakers, from Romania and abroad. A number of experts, young oncologists and leaders of the oncology societies shared their views and opinions about the present and future oncology directions in the country, Europe and globally. These can be found at https://ecancer.org/en/conference/1288-romanias-national-congress-of-oncology-2020.

## Conflicts of interest

The authors have declared that they have no conflicts of interest.

## Funding statement

The authors received no specific funding for this work.

